# Epigenetics of cervical cancer. An overview and therapeutic perspectives

**DOI:** 10.1186/1476-4598-4-38

**Published:** 2005-10-25

**Authors:** Alfonso Dueñas-González, Marcela Lizano, Myrna Candelaria, Lucely Cetina, Claudia Arce, Eduardo Cervera

**Affiliations:** 1 Unidad de Investigación Biomédica en Cáncer, Instituto Nacional de Cancerología/Instituto de Investigaciones Biomédicas (INCan/IIB), Universidad Nacional Autónoma de Mexico (UNAM), Mexico City. Mexico; 2 Division of Clinical Research, Instituto Nacional de Cancerología (INCan), Mexico City, Mexico

## Abstract

Cervical cancer remains one of the greatest killers of women worldwide. It is difficult to foresee a dramatic increase in cure rate even with the most optimal combination of cytotoxic drugs, surgery, and radiation; therefore, testing of molecular targeted therapies against this malignancy is highly desirable. A number of epigenetic alterations occur during all stages of cervical carcinogenesis in both human papillomavirus and host cellular genomes, which include global DNA hypomethylation, hypermetylation of key tumor suppressor genes, and histone modifications. The reversible nature of epigenetic changes constitutes a target for transcriptional therapies, namely DNA methylation and histone deacetylase inhibitors. To date, studies in patients with cervical cancer have demonstrated the feasibility of reactivating the expression of hypermethylated and silenced tumor suppressor genes as well as the hyperacetylating and inhibitory effect upon histone deacetylase activity in tumor tissues after treatment with demethylating and histone deacetylase inhibitors. In addition, detection of epigenetic changes in cytological smears, serum DNA, and peripheral blood are of potential interest for development of novel biomolecular markers for early detection, prediction of response, and prognosis.

## Overview of cervical cancer

### Epidemiology and treatment

Cervical cancer remains one of the greatest killers of women worldwide. According to Globocan 2000, it is estimated that in 2000 the numbers of patients diagnosed with and those who died from this disease were 470,606 and 233,372, respectively [1]. It is remarkable that these rates occur despite the fact that cervical cancer is a model for early detection due to its long and relatively well-known natural history, which offers an excellent opportunity for its detection before lesions become invasive [2].

Cervical cancer is currently staged clinically according the International Federation of Gynecology and Obststrics (FIGO) guidelines. In terms of treatment, invasive disease can be divided into three main groups: 1) early stage going from microinvasive disease IA1, IA2 to macroscopic disease confined to cervix and measuring <4 cm, IB1; 2) locally advanced FIGO stages IB2-IVA, and 3) IVB and recurrent disease [3].

### Treatment of early stages

The recommended treatment for IA1 patients is either a local procedure such as conization or total hysterectomy depending on the patient's desire to remain fertile, whereas for IA2 patients the recommended procedure is a radical one including pelvic lymphadenectomy. On average, 8% of cases shows positive pelvic lymph nodes. As many women at this disease stage deserve to preserve fertility, radical trachelectomy is becoming an option for these patients. The same can apply for IB1 patients. In early cases that are surgically treated, the presence in the surgical specimen of a combination of intermediate-risk factors (vascular and lymphatic permeation, tumor size >2 cm, and deep cervical stroma invasion) or high-risk factors (positive pelvic lymph nodes, parametrial infiltration, and positive surgical margins) dictates use of adjuvant radiation or chemoradiation respectively. As a group, the prognosis of early-stage cases is fairly good with 5-year survival exceeding 90% [4,5]

### Treatment of locally advanced stages

Results of treatment for these patients are far from optimal. In this regard, treatment of locally advanced cervical cancer has experienced no major changes for nearly 80 years during which exclusive radiation was considered the standard of care; thus, 5-year survival for stages IB2, IIB, IIIB, and IVA are 72.2, 63.7, 41.7, and 16.4%, respectively, according the 1998 Annual Report on the Results of Treatment in Gynaecological Cancer [6]. The lengthy permanence of this unimodal treatment was due, on the one hand, to the classical concept that cervical cancer is a disease that progresses in an orderly fashion (local, then regional, and at the very last, systemic); therefore, it could be effectively treated with a local modality such as radiation instead of a systemic modality such as chemotherapy. On the other hand, the role of surgery for locally advanced cases failed to treat the disease successfully by radical surgical procedures [7]. Over the last 20 years, however, an increasingly number of trials that incorporate either chemotherapy and/or surgery with radiation (neoadjuvant chemotherapy followed by radiation, neoadjuvant chemotherapy followed by surgery plus minus adjuvant radiation, and concurrent chemoradiation) have been performed in an attempt to improve treatment results. Radiation concomitant with cisplatin-based chemotherapy is considered the current standard of treatment. This combined modality produces an absolute increase in 5-year survival of 12% as compared with radiation alone. On the other hand, neoadjuvant chemotherapy when followed by surgery – but not when followed by radiation – yields a 15% increase in absolute 5-year survival. These data emerged from three meta-analyses of the literature based on individual patient analysis [8,9].

### Treatment of IVB and recurrent disease

Patients with cervical cancer may present at diagnosis with distant metastases (stage IVB) or have, after primary treatment, pelvic recurrence, distant metastases, or a combination of both. Recurrence rates vary from up to 20% to 70% in early stages and locally advanced disease, respectively [10,11] and the majority of recurrences occur within 2 years of diagnosis. At this stage, cervical cancer is even more difficult to treat because this clinical situation is the result of a more malignant phenotype resulting from accumulation of genetic defects during tumor progression and previous therapies; thus, the tumor at this stage is commonly resistant to chemotherapy. Moreover, these patients frequently have a poor performance status that limits use of agressive chemotherapy and the majority of patients die as a result of uncontrolled disease. In this setting, the few patients who recur after initial surgical treatment can be salvaged with concurrent chemoradiation if the disease is local or locoregional [12]. Those who receive primary radiation or chemoradiation and have pelvic disease can be offered an ultraradical procedure such as pelvic exenteration; nonetheless, this procedure is currently limited to patients with small and central tumors that in these situations, pelvic exenteration may offer 5-year survival for up to 50% of patients [13]. Although some efforts have been devoted to extending the exenterative procedures to patients with higher disease burdens by use of intraoperative radiation [14], laterally extended pelvic exenteration [15], or pre-exenterative chemotherapy [16] none of these options are widely used. Unfortunately, patients with IVB and those with distant metastases – with or without pelvic relapse – have no option other than systemic chemotherapy that in this setting has limited value; cisplatin is the most active single agent [17] and more recently in combination with topotecan has shown a modest increase in time to progression and median survival as compared with single agent cisplatin. In any case, median survival remains between 6 and 12 months [18].

## Molecular pathogenesis of cervical cancer

### Human papillomavirus

Current experimental and epidemiologic information undoubtedly points to the human papillomavirus (HPV) as the primary causal agent in development of cervical carcinoma. Therefore, the study of its carcinogenic role continues to represent the mainstream research on the molecular biology of cervical cancer, with the idea that prophylactic and therapeutic applications of knowledge from this field could benefit millions of women afflicted, or at risk to be afflicted, with HPV-induced cervical cancer.

HPV classification is predicted on DNA sequence homology. At least 200 types have been identified and these have been classified into 16 groups [19]. Genital HPVs are classified according to their potential to induce malignant transformation as follows: high-risk types (16, 18, 31, 33, 35, 39, 45, 51, 52, 56, 58, 59, 68, 73, and 82); probable high-risk types (26, 53, and 66), and low-risk types (6, 11, 40, 42, 43, 44, 54, 61, 70, 72, 81, and CP6108) [20]. Among high-risk strains, HPV 16 and 18 are those most closely associated with cervical carcinoma and are found in >50% and 20% of squamous cell carcinomas, respectively [21].

A large body of knowledge supports the view that high-risk HPV types (HR-HPV) have the ability to transform cells into a malignant phenotype. Nevertheless, only a minority of cervical lesions infected with HR-HPV inevitably progress to cervical carcinoma, as indicated by frequent spontaneous clearance of HPV infection and the long delay between onset of persistent infection and emergence of the malignancy. For that reason, studies have been focused on analyzing the participation of possible viral and cellular factors governing HPV-induced malignancy.

### HPV structure

Human papillomaviruses (HPV) belong to the Papovaviridae family. They consist of a 72-capsomere capside containing the viral genome. Capsomers are composed of two structural proteins: the 57 kD late protein L1, which accounts for 80% of the viral particle, and the 43–53 kD minor capside protein L2. The HPV genome consists of eight kilobasepairs (Kbp) and is a double-stranded DNA molecule. Arrangement of the 8–10 open reading frames (ORFs) within the genome is similar in all papillomavirus types, and partly overlapping ORFs are arranged on a sole DNA strand. The genome can be divided into three regions: the long control region (LCR) without coding potential; the region of early proteins (E1–E8), and the region of late proteins (L1 and L2) [22].

### Early gene products

E1 and E2 encode proteins that are vital for extrachromosomal DNA replication and completion of the viral life cycle [23]. A hallmark of HPV-associated cervical carcinoma is loss of the expression of viral E2 protein [24]. A fusion product consisting of the small E8 ORF with part of the E2 protein has been described. This fusion protein is able to repress viral DNA replication as well as transcription, and is therefore believed to play a major role in the maintenance of viral latency observed in the basal cells of infected epithelium [25,26]. The E4 protein is expressed in the later stages of infection when complete virions are being assembled, and is not known to have transforming properties; however, it is considered to play an important role in the maturation and replication of the virus [27]. The E5 in open reading frame is often deleted in cervical carcinoma cells, indicating that it might not be essential in maintaining the malignant transformation of the host cell. Nevertheless, it has been reported that E5 protein possesses a weak transforming activity [28].

### E6 and E7 proteins

E6 and E7 are the most important oncogenic proteins. Transcription of E6 and E7 genes has been observed to occur always in cervical carcinomas, being the first indication of a main role of these viral genes in HPV-associated tumorigenesis. The immortalization and transforming potential of E6 and E7 proteins have been demonstrated in tissue culture and in experimental animal models [29]. From the studies of E6-p53 and E7-pRb models, numerous actions have been identified of viral gene products on cellular proteins. Therefore, several findings hint at possible ways by which HPV-infected cells may escape controls governing cell growth and proliferation.

The E6 protein of high-risk HPV anogenital types shows weak oncogenic potential in the majority of established cell lines, and cooperation with E7 protein is required for full transforming capacity. Discovery of the inactivation of the tumor suppressor genes p53 and pRB by E6 and E7 oncoproteins provided a basic explanation of how high-risk HPV types induce their oncogenic effects on cervical cells [30]. E6 has many interactions with cellular proteins; nevertheless, its key action is inhibition of the function of tumor suppressor protein p53 by enhancing its degradation through the ubiquitin pathway [31,32]. To inhibit p53 function, E6 requires a cellular protein called E6-associated protein (E6AP). In non-infected cells, ubiquitin-mediated degradation of p53 is triggered by the mdm-2 protein, while in HR HPV-infected cells the E6-E6AP complex replaces mdm-2 in control of cellular p53 levels. This shift shortens the p53 half-life and reduces its levels in cervical carcinoma cells to less than one half of the level found in normal ephithelial cells [33]. It is known that increase in p53 levels plays a critical role in the induction of genes that results in cell cycle arrest [34], allowing repair of damaged DNA or activation of apoptotic pathways [35]. Therefore, cells expressing E6 maintain low levels of functional p53, altering normal response to DNA damage and favoring accumulation of genomic mutations. Binding of the E7 oncoprotein on pRB provides a complementary function. Binding releases transcription factor E2F that activates expression of genes that stimulate DNA synthesis in the cell. If earlier E6 action had freed the same cell from p53 control, that cell survives into the S phase with damaged DNA and, through E7 action, is able to replicate the HPV DNA [36]. Oncogenic properties of E6 and E7, as well as their effects on p53 and pRB, have provided the general basis for further investigations of the role of HPV in carcinogenesis in the HPV-infected cervix. Research in the action of the two oncoproteins have shown how they subvert key cell cycle and regulatory processes such as cyclins, cyclin-dependant kinases (CDKs), and cyclin-dependant kinase inhibitors (CDIs), among other interactions, to transform and immortalize the host cell [37].

### DNA integration

HVP DNA is usually extrachromosomal or episomal in benign cervical precursor lesions. Cancer tissues may contain both episomal and integrated HPV DNAs at the same time, although integration appears to occur more frequently in HPV 18- than in HPV 16-associated cervical cancer [28]. During HPV DNA integration, the viral genome usually breaks in the E1/E2 region. The break generally leads to loss of the E1 and E2 regions. Loss of E2 results in uncontrolled and increased expression of E6 and E7 oncogenic proteins. Increased expression of E6 and E7, meanwhile, has been observed to lead to malignant transformation of host cells and to tumor formation [38]. HPV viral integration into host genome DNA is associated with progression from polyclonal to monoclonal status in cervical intraepithelial neoplasia (CIN), and these events play a fundamental role in the progression from low- to high-grade cervical neoplasia [39].

## Epigenetics

Epigenetics can be defined as the study of genoma function that is contained outside of DNA itself and by means of which stable alterations in gene expression are set. Epigenetics is a well-established phenomenon that plays a major role in a diversity of biological processes such as embryonic development, cancer biology, and immune system response, among many others. The two most widely studied epigenetic changes are DNA methylation and histone acetylation; however, the picture is much more complicated than this, with new players coming onto the scene such as the RNA interference phenomenon, which has proven to be implicated in transcriptional silencing through small duplex RNA molecules that recruit silencing complexes to the chromatin [40,41,22].

### DNA methylation

DNA methylation is a covalent chemical modification that occurs at the cytosine ring, resulting in the addition of a methyl (CH3) group at the carbon 5 position. According to the fact that DNA is made up of four bases and that therefore 16 possible dinucleotide combinations can occur, the CpG dinucleotide should have a frequency of 6%. However, the actual presence is only 5–10% of its predicted frequency. The human genome is not methylated uniformly and contains regions of unmethylated segments interspersed with methylated region. In contrast to the remainder of the genome, smaller regions of DNA, called CpG islands – and ranging from 0.5–5 kb and occurring on average every 100 kb – have distinctive properties. These regions are unmethylated, GC-rich (60–70%), have a ratio of CpG to GpC of at least 0.6, and thus do show no suppression of dinucleotide CpG frequency. Approximately one half of all genes in humans have CpG islands, and these are present in both housekeeping genes and genes with tissue-specific patterns of expression [43-46]. At least three functional DNA methyltransferases (DNMTs) have been identified; the most abundant is DNMT1, which preferentially methylates hemi-methylated DNA. DNTM1 localizes to replication foci and is responsible for maintaining proper methylation levels during replication and possibly DNA repair [47,48]. Other known functional methyltransferases are DNMT3a and DNMT3b, which are responsible for *de novo *methylation during embryogenesis [49]. DNMT3a and DNMT3b have equal preferences for hemi-methylated and non-methylated DNA, and thus have been classified as *de novo *methyltransferases[50]. In addition to DNMTs, the machinery of methylation includes demethylases, methylation centers triggering DNA methylation, and methylation protection centers [51]. The effect of DNA methylation on gene transcription can only be seen in the context of chromatin remodeling players. DNA methylation can directly interfere with transcriptional factor binding and thus inhibit replication [52], in addition to the ability of DNA methyltransferases DNMT1, DNMT3a, and DNMT3b to repress transcription in a methylation-independent manner [53]. Methyl-CpG binding proteins, which can recognize methylated DNA, have been shown to associate with large protein complexes containing HDACs and chromatin-remodelling activities, and it has also been suggested that DNA methylation could produce gene silencing by methyl binding domain proteins that recruit histone methyltransferases, which methylate lysine 9 in histone H3 and subsequently repress gene transcription [54]; as a result, histones are deacetylated and gene transcription is most often repressed.

### Histones and post-translational modifications

How double-strand DNA is packaged into the dynamic structure of chromatin is crucial for the process of transcriptional control by regulating transcription factor accessibility to DNA regulatory sequences. Chromatin is constituted of nucleosomes, which are comprised of 146 base pairs of DNA wrapped around a core of two copies each of H2A, H2B, H3, and H4 histone proteins. These proteins suffer post-translational modifications that play a prominent role in gene expression regulation and signal transduction pathways such as methylation, acetylation, ubiquitination, phosphorylation, and sumoylation, which determine chromatin architecture and ultimately gene transcription [57].

The most widely studied modification is acetylation. Addition of charge-neutralizing acetyl groups to lysine residues on histones disrupts interactions with DNA, resulting in chromatin decompactation, greater access of DNA to transcription factors, and the presence of a transcriptionally active genomic locus. This post-translational modification depends on the net local balance between activities of histone acetyltransferase (HAT) and histone deacetylase (HDAC). In general, increased levels of histone acetylation (hyperacetylation) are found in more decondensed euchromatin, whereas decreased levels of acetylation (hypoacetylation) are characteristic of more condensed heterochromatin [58]. However, this mechanical model is an oversimplification of how gene transcription is regulated as additional histone modifications influence transcription [59].

Histone methylation can occur on lysine and arginine residues, giving the cell another layer of regulatory options, for example, lysine 9 in histone H3. It is currently known that histone arginine methylation is more dynamic, correlating well with gene activation and its loss from target arginines in H3 and H4 with gene inactivation. In contrast, lysine methylation appears to be a more stable mark. In this sense, methylation of lysine 4 in histone H3 correlates with gene activation, whereas methylation of lysines 9 and 27 in histone H3 correlates with repression [61-63]. Phosphorylation is another important and long-appreciated histone modification often associated with chromosome/chromatin condensation that includes mitosis, meiosis, apoptosis, and DNA damage, events regulated by different histone kinases (for example, members of the Aurora/AIK family [64-66]. Along with this post-translational modification of histone proteins, sequence-specific DNA binding by transcription factors and other protein remodeling factors determine a histone code for gene-specific transcriptional control that may dictate which modification or specific combinations of histone modifications can affect distinct downstream events by altering chromatin structure and/or generating a binding platform for protein effectors that can specifically recognize the modification and initiate gene transcription or repression [67].

## Epigenetic alterations in cancer

Because of the close interplay between DNA methylation and histone modifications, it is expected that both mechanisms are operating in disease processes such as cancer; nonetheless, for the majority of tumor types the epigenetic defects could be just one of the many molecular cell alterations that lead to the malignant phenotype.

### DNA methylation and cancer

Abnormalities in DNA methylation have long been associated with cancer. Both hypo- and hypermethylation play a prominent role in carcinogenesis, and their contribution shows scarcely defined boundaries. It has long been known that in cancer cells both alterations coexist: malignant tumors show global hypomethylation and regional hypermethylation. Whether one must precede the other or whether both should start at the same time remains to be elucidated. In terms of carcinogenesis, the first observations in fact were done on hypomethylation [69]; later, the discovery of regional hypermethylation as a means to silence the tumor suppressor genes expression gained the most attention [70].

### Hypermethylation and gene silencing

Observations that tumor suppressor genes can be inactivated not only through structural changes (mutation, deletion) but also by lack of expression due to promoter hypermethylation positioned tumor suppressor gene epigenetic silencing as a well-established oncogenic process [71]. The first suppressor gene known to be hypermethylated and silenced was *RB *[72], which was followed by multiple publications describing similar findings for a variety of tumor suppressor genes, among them *p16*, *MLH1*, *VHL*, and *E-cadherin *[73].

Whether gene promoter hypermetylation is the cause or consequence for the tumor suppressor gene silencing is still a matter of controversy; nevertheless, these views are not mutually exclusive. That DNA methylation is causal has been shown by the ability of diverse pharmacologic compounds and molecular techniques to reactivate gene expression upon inhibition of DNA methylation in cancer cells [74].

On the other hand, other findings suggest that hypermethylation-induced gene silencing could be secondary to changes that determine gene expression, such as chromatin modification, so that methylation helps to maintain the silenced status of the gene. Strong support for the second view came from experiments showing that methylation of histone H3 lysine 9 – that is, chromatin modification – occurred, along with re-silencing of *p16 *in absence of DNA methylation in cells in which *p16 *had previously been activated by knocked out of DNA methyltransferase [75] and by data demonstrating *p16 *silencing in mammary epithelial cells that had escaped senescense and had demethylated the promoter [76].

### Hypomethylation and gene activation

It is known that tumor cells have global DNA hypomethylation that can be as high as 60% less than their normal counterparts [77]. This hypomethylation occurs mainly in the body of genes (coding regions and introns), as well as in pericentromeric regions of chromosomes rich in repetitive DNA sequences [79]. Interestingly, hypomethylation is progressive from premalignant conditions to fully developed malignancies [80]. The main mechanisms put forward in attempting to explain cancer causation by hypomethylation include chromosome instability and reactivation of transposable elements and/or inappropriate gene activation [81]

There are two pieces of convincing evidence linking hypomethylation with chromosomal instability. The congenital disorder ICFs syndrome (immunodeficiency, chromosomal instability, and facial anomalies) caused by mutations at *DNMT3b *demonstrates loss of methylation in classical satellite DNA and mitogen-inducible formation of bizarre multiradial chromosomes that contain arms from chromosomes 1 and 16 [82]. This disorder, however, is not associated with cancer, but common somatic tumors such as breast, ovarian, and other epithelial tumors commonly have unbalanced chromosomal translocations with breakpoints in the pericentromeric DNA of chromosomes 1 and 16 [83]. In mouse models with an inactivated allele of *NF1 *and *p53 *genes, introduction of a hypomorphic *DNMT1 *allele caused a 2.2-fold increase in LOH frequency [84].

Finally, some reports have stressed the fact that many CpG islands are normally methylated in somatic tissues [85], and that demethylation could lead to activation of nearby genes such as *HRAS*. Indeed, experimental demonstration exists that hypomethylation leads to activation of genes important for cancer development, including promoter CpG demethylation and overexpression of *14-3-3sigma*, *maspin*, *heparanase*, and *S100A4 *in several tumor types [86-88]. The question here is whether over-expression was indeed caused by hypomethylation or whether promoters are hypomethylated secondary to its high transcriptional activity. There are data showing that the sole hypomethylation as achieved by pharmacologic means is not sufficient to activate gene expression. In this context, some genes are not permisive for expression; this means that despite the fact that methylation is relieved the necessary ancillary factors to activate transcription are not present. Others are permissive and therefore reactivated by demetylation, whereas for others hypomethylation does not affect their levels of expression but can be over-expressed due to activation of signalling pathways known to activate them [89].

### Chromatin and cancer

All classical genetic alterations – for instance, mutations in oncogenes or tumor suppressor genes of malignant cells – eventually affect gene transcription (mutant *Ras*, *HER2 *amplifications), or are transcription factors in themselves (*c-myc*, *p53*). It is therefore not surprising that the machinery of transcription control could be directly involved in the carcinogenesis process. Although the complex nature of the regulation of transcription is clear, certainly a disruption in the balance of activities of enzymes in charge of maintaining histone acetylation status is expected to occur in cancer. Among histone acetylases, the coding genes of p300/CBP have been found altered in some neoplasms. Mutations have been observed in epithelial tumors such as lung, esophageal, ovarian, and gastric tumors [90-93]. Chromosomal translocations involving CBP or p300 that in turn disrupt transcription by its fusion with partern genes are well-described molecular defects leading to hematologic malignancies such as some forms of acute myeloid leukemias [94,95]. Histone deacetylase activity leading to aberrantly repressed transcription was one of the first described leukemogenesis events. In acute promyelocytic leukemia, *PML*-*RAR*α translocation generates a fusion gene product that represses transcription by associating with a co-repressor complex that contains HDAC activity [96]. Similar mechanisms account for other types of leukemia and lymphomas such as *AML1-ETO *and *LAZ3/*BCL6, respectively [97,98].

Despite the fact that participation of DNA methylation and chromatin in the carcinogenic process is unquestionable, it must be borne in mind that the split of epigenetics and genetics as separate types of defects in cancer is very artificial. In fact, according to the definition of epigenetics as genetic information not contained in the DNA sequence itself, current evidence demonstrates that primary genetic defects (mutations in genes with no known primarily methylating or chromatin-modifying activity such as growth factor receptors, adhesion molecules, etc,, or mutations in genes that in themselves affect DNA methylation or chromatin such as DNMTs or HAT/HDAC) are those leading to altered DNA methylation and chromatin changes. Demonstration that exogenous or endogenous carcinogens without causing primarily gene mutations lead to epigenetic abnormalities should prove that epigenetics is by itself one of the carcinogenic steps.

## Epigenetic alterations in cervical cancer

Because infection with high-risk types of human papillomavirus is needed for cervical cancer development, it is important to consider the epigenetic changes occurring in the viral genome that can influence the virus-driven carcinogenic process as well as epigenetic changes in the host genome. Table 1 summarizes epigenetic alterations found in cervical cancer.

**Table 1:** Main epigenetic alterations in cervical cancer

**Alteration**	**Meaning**
**HPV-related**	
Methylation of HPV-E2 binding sites	De-repression of E6 and E7 HPV oncoproeteins?
Methyation at HPV-E6 and E7 LCR	Cause or consequence of E6/E7 over-expression?
E6 and/or E7 interaction with DNMTs?	Silencing of cellular tumor suppressor genes?
Interaction between E7 with HDACs	Aid for cell transformation
Interaction between E6 with HATs	Aid for cell transformation
	
**Host cell-related**	
Regional DNA hypermethylation	Silencing of tumor suppressor genes
Global DNA hypomethylation	Genomic instability?, oncogen over-expression?
Abnormal pattern of chromatin	Unknown
Loss of imprinting at H19/IGF2 loci	Tumor progression?
H3 hyper-phosphorylation & acetylation	Associated with carcinogenesis Progression

### HPV and methylation

The realization that viral infections, by insertion of viral genes into host genomes, can trigger host defense mechanisms such methylation machinery activation has aroused interest in the study of epigenetic events occurring in both virus and host genomes [99]. Human genomes harbor DNA sequences resembling retroviral long terminal repeats and the transposable elements, and indeed there are indications that under some situations inappropiate "activation" of these normally silenced sequences could play a role in the carcinogenic process [100]. In addition, it is also established that some viruses can find ways to adapt different tactics to regulate expression of their genes through modulation of DNA methylation; thus, a virus may silence activation of its genes in a manner that favors establishment of persistent infection and evades the host immune defense [101]. In addition to this, viral oncoproteins can possess the ability to modulate directly or indirectly the methylation machinery in order to silence cellular genes that could interfere with its tumor promoting actions. A very illustrative example of this is how the Epstein-Barr virus oncogene product, latent membrane protein 1, induces downregulation of *E-cadherin *gene expression via activation of DNA methyltransferases [102].

The role of HPV genome DNA hypermetylation has of late been the subject of study. One of the first indications of the importance of DNA methylation and viral gene expression came from studies of cell transfection with HPV-16 *in-vitro *methylated genomes, demonstrating that under these circumstances DNA is transcriptionally repressed [103]. In SiHa and CasKy cell lines that harbor HPV-16 and have a couple of and multiple viral genome copies, respectively, a conserved profile of CpG hyper and hypomethylation was found by using scanning with the restriction enzyme McfBC. Hypermethylation was found in genomic segments overlying late genes, while the long control region and the E6 and E7 oncogenes were unmethylated in SiHa cells. Interestingly, evaluation of smears of normal, precursor, and invasive lesion smears of 81 patients showed that as lesion severity increases, there is progressive hypomethylation on these LCR and E6 gene regions; thus, hypermethylation was found in 52% of smears from asymptomatic women, in 21.7% of preinvasive lesions, and only in 6.1% of invasive-case smears. These findings lead the authors to postulate that neoplastic transformation can be suppressed by gene hypermethylation, whereas hypometylation accompanies or causes cancer progression [104]. These findings however, were not totally coincident in another study that studied L1 and LCR regions by bisulfite modification in 115 clinical samples. First, high heterogeneity on methylation status was noted among patients and even in different samples of the same patient. As expected, methylation frequency was ca. 30% in the L1 region and lower in other positions, particularly at a CpG site located in the linker between two nucleosomes positioned over HPV-16 enhancer and promoter. However, methylation at most sites was consistently higher in carcinomas as compared with dysplasia, possibly related to the tandem repetition and chromosomal integration that occurs in invasive lesions [105]. In another study performed in two HPV-18 cervical cancer cell lines, HeLa and C4-1, and clinical samples, there was also clonal heterogeneity in the methylation status of the different regions analyzed. When it came to clinical sample analysis, there was complete or partial HPV-enhancer methylation in three of six tumors and complete demethylation in eight smears from asymptomatic patients. Likewise, promoter methylation was found in three of six cancers and in four of six smears [106]. The latter two studies suggest that methylation status of viral oncogenes in lesions perhaps is perhaps solely the result of their transcriptional activity level and not a causal event for neoplastic progression.

Further data on the influence of DNA methylation in the HPV life cycle comes from another study that focused on methylation of *E2*, the early gene that contributes to multiple biological processes including viral transcription and viral DNA replication. It has been shown that the capacity of E2 protein to bind E2BS *in vitro *is inhibited by methylation of these cytosines [107]. Kim et al. performed a methylation analysis by bisulfite modification of *E2 *binding site within LCR in DNA isolated from an immortalized epithelial cell line isolated from an HPV 16-infected patient and demonstrated that this region is selectively hypomethylated in the highly differentiated cell populations, whereas poorly differentiated basal-like cells were heavily methylated particularly in E2 binding sites. These observations may indicate that the methylation state of the viral genome, and particular that of E2BSs, may vary during the viral life cycle, providing a novel means for modulating E2 function as infection progresses [108]. It will be of major interest to analyze human papillomavirus oncogene expression in cervical tumors before and after treatment of patients with DNA methylation inhibitors.

### Hypermethylated genes in cervical cancer

There are numerous reports demonstrating that tumor suppressor genes belonging to nearly every cancer pathway or function category have silenced or diminished their expression due to abnormal promoter hypermethylation in cervical carcinoma (Table 2).

**Table 2:** Tumor suppressor genes hypermethylated in invasive cervical cancer

**Gene**	**Rate**	**Function**	**Reference**
DcR1/DcR2	100%	Apoptosis	113
hTERT	57%	Apoptosis	122
p73	39%	Apoptosis	129
p16	8–42%	Cell-cycle	130–136
PTEN	58%	WNT-pathway	142
E-cadherin	28–80.5%	WNT-pathway	143–145
APC	11–94%	WNT-pathway	133,135,136
MGMT	5–81%	DNA repair	133,134,136,144
FANCF	30%	FA-BRAC pathway	161
BRAC1	6.1%	FA-BRAC pathway	133
hMLH1	5%	Mismatch repair	134
RASSF1A	0–45%	Negative ras-effector	144,172–174
DAPK	45–100%	Metastasis/cell death	133,135,136,144
TSLC1	58–65%	Tumor suppressor	179,180
FHIT	11–88%	DNA repair?/cell death?	133,134,135,136,144
HIC1	18–45%	Transcription factor	133,135
RARβ	33–66%	Cell differentiation	133,136,200,201
TIMP2/TIMP3	47%/1–10%	Tissue inhibitor MTs	144,202,203
Caveolin-1	6%	Caveolae membrane	205
ER α	25%	Steroid hormone receptor	136

### Apoptosis-related genes

It is now established that failure of cells to undergo apoptosis is crucial for cancer development and progression, but most importantly this phenomenon participates in intrinsic or acquired resistance of cancer cells to chemotherapy and radiation. Identification of points in the apoptotic pathway at which dysregulation occurs would potentially open up new therapeutic opportunities in situations where conventional cancer treatments fail. One of the first indications of the role of methylation for inactivation of key apoptotic genes came from the study showing that *Apaf-1 *was silenced in melanoma instead of being lost or mutated [109].

Studies analyzing apoptosis-related genes that can be inactivated by methylation in cervical cancer are limited. One study has shown that decoy receptors DcR1 and DcR2 can be the target for abnormal methylation that leads to their silencing [110]. These molecules are members of the tumor necrosis factor receptor superfamily, which includes TNFR1, *Fas*, and the decoy receptors for TRAIL. Upon engagement by their respective ligands, TNFR1 and FAS recruit adaptor molecules and activate a cascade of caspases. Death-inducing decoy receptors *DR4 *and *DR5 *and *DcR1 *and *DcR2 *are structurally related; nonetheless, *DcR1 *completely lacks the intra-cellular death domain and *DcR2 *contains a truncated, nonfunctional death domain and appears unable to induce apoptosis. Hence, *DcR1 *or *DcR2 *have been postulated to function as oncogenes because of their postulated anti-apoptotic effects [111,112]. In cervical carcinoma, a study has found that all 50 cases analyzed had methylation of either DcR1 and/or DcR2 [113], suggesting that cervical cancer cells, by downregulating decoy receptor expression, obtain a growth advantage.

Telomerase activation is a critical element in cellular immortalization and cancer. The end of the chromosome, the telomere, plays a critical role in chromosome structure and function. A certain length of the telomere is important for cell division, and the telomere may serve as a "mitotic clock" for cell proliferation. Normal human somatic cells express low or undetectable telomerase activity, whereas in immortal eukaryotic cells as well as in cancer cells telomerase activity increase is apparently necessary to ensure proliferation. Telomerase is a ribonucleoprotein comprising an RNA template, the telomerase-associated protein, and the catalytic subunit (*hTERT*) [114,115]. Telomerase activity has been demonstrated in various types of gynecologic cancers [116]. Data on *hTERT *expression in cervical cancer has revealed that 0–33% of normal cervices exhibited *hTERT *mRNA expression, whereas 80–100% of cervical cancers showed *hTERT *expression [117-120] The fact that the *hTERT *gene promoter has a CpG island and high overall GC content suggests a possible role for methylation in regulation of *hTERT *gene expression; however, the relationship between gene promoter and expression is unclear for this gene. Despite it is expected that hypermethylation decreases gene expression, a study has found a correlation between reduced expression and catalytic subunit activity with demethylation [121]. This may explain what was found with regard to better prognosis of patients with cervical cancer whose tumors lack *hTERT *methylation [122].

The p53 pathway responds to stresses that can disrupt the fidelity of DNA replication and cell division, resulting in activation of the p53 protein as a transcription factor that initiates either growth arrest or apoptosis. This apoptosis pathway is disrupted in the majority of human cancers by downregulation or loss of p14^ARF^, upregulation of MDM2, or mutation of p53 [123,124]. However, this pathway, by virtue of its multiple positive and negative feedback loops, can be the target of aberrant methylation in some of their components. *p73 *is a member of the *p53 *family that encodes two different proteins expressed under the control of two independent promoters and that have opposite activities: the transcriptionally active full-length *TAp73 *and the NH_2_-terminally truncated dominant-negative *Np73 *[125]. *TAp73 *has been reported as involved in cellular response to DNA damage induced by radiation and chemotherapeutic agents and when it is overexpressed in cells, it activates transcription of *p53*-responsive genes such as *p21*, *Bax*, *Mdm2*, and *GADD45 *and inhibits cell growth in a *p53*-like manner by inducing apoptosis [126,127]. It has been reported that *p73 *transcription can be regulated by the promoter and exon 1, which is rich in CpG dinucleotides [128], and its transcriptional silencing through methylation is a common event in some leukemias, lymphomas, and brain tumors, as well as in ovarian cell lines but not in breast, renal, and colon cancers. A recent study found that epigenetic modification of *p73 *via CpG-island hypermethylation represents a critical alternative mechanism for inactivation of this gene in cervical cancer and high incidence of *p73 *hypermethylation (38.8%) in cervical cancer but not in controls; in addition, its methylation was correlated with loss of its p73 expression. Importantly, radioresistant cancer samples had significantly higher methylation rate than radiosensitive cancer samples, and *in vitro *demethylation successfully restored p73 expression in cervical cancer cell lines previously found to have methylated *p73 *and lack of p73 mRNA and protein expression [129].

### Cell cycle-related genes

It is well-established that cancer cells evolve in part by overriding normal cell-cycle regulation. Normal cell cycle progression relies on the cell's ability to translate extracellular signals such as those produced by growth factor receptor stimulation and extracellular matrices to efficiently replicate DNA and divide. Proper cell- cycle regulation is essential for cells and requires a number of players, among them cyclin-dependent kinases and their binding partners along with natural inhibitory molecules such as p16, Rb, and p15 that play an essential role. Within this class, the p16 gene has been one of the most studied in cervical cancer. Aberrant methylation of the *p16 *gene occurs early within tumor cell populations in both *in situ *and invasive tumors at frequencies that vary from 10 up to 100% [130-136]. As the cell cycle is primarily deregulated by the HPV, the molecular contribution of p16 inactivation is unclear; however, the fact that this not only is a very early event in cervical carcinogenesis but is more frequently methylated in advanced tumors [132] suggests that its reactivation could have therapeutic value. Despite the fact that Rb and p15 are known to be inactivated by methylation in other tumors, no reports exist on cervical tumors.

### WNT pathway

The Wnt signaling pathway, named for its most upstream ligands, the Wnts, is involved in various differentiation events during embryonic development and leads to tumor formation when aberrantly activated. Within this pathway, there are a number of participating molecules, and the pathway is regulated by a multiprotein complex consisting of, among others, members of β-catenin, the key component, adenomatous polyposis coli APC, Axin, and GSK-3β. [137]. In the absence of Wnt stimulation, β-catenin accumulates in cytosol to then be translocated to nucleus, leading to transcription of target genes. This pathway is also involved in calcium-dependent cell adhesion by virtue of the interaction between β-catenin and cadherin [138]. There are mutations in APC, another key regulator of the pathway that promotes β-catenin proteolysis and reduces its transcriptional activity. PTEN is a lipid and protein phosphatase that is a negative regulator of phosphatidylinositol 3 (PI-3) kinase-dependent signaling and influences the WNT pathway by hindering activation of integrin-linked kinase (ILK), which inhibits GSK-3 β and thereby causes accumulation of β-catenin [139]. The WNT signaling pathway is the most frequently altered pathwayin the majority of cancers; for instance, it has been demonstrated that nearly all colorectal cancers have at least one activating mutation in this pathway [140]. As such, individual components of the pathway can be targeted by epigenetic inactivation. A recent study analyzing 310 colorectal carcinomas for eight members of the signaling cascade, including APC, β-catenin, AXIN2, TCF4, WISP3, E-cadherin, and PTEN. Hypermethylation on E-cadherin and APC were found at frequencies between 36 and 42% [141].

Studies on cervical cancer have uncovered that hypermethylation of these genes is not uncommon. A study in 62 cases of squamous cell carcinomas showed that while PTEN mutations were absent, promoter methylation was found in 58% of cases. Interestingly, patients with persistent disease or patients who died of the disease had a significantly higher percentage of PTEN methylation than those without evidence of recurrence. Multivariate Cox regression models confirmed PTEN was an important significant predictor for both total and disease-free survival after controlling age, pathologic grade, and clinical stage [142]. Inactivation of the cadherin-mediated cell adhesion system caused by aberrant methylation is a common finding in human cancers. Methylation frequency of *E-cadherin *in cervical cancer varies from between 28 and 80.5% [143-145] and appears to have prognostic significance, cases with no promoter methylation having a better outcome in univariate and multivariate analyses [146]. Mutations are the main mechanism of inactivation for *APC*, particularly for colon and other tumors from the gastrointestinal tract. However, *APC *promoter hypermethylation occurs in other cancers. Frequencies of methylation in 208 primary human tumors of multiple origins were as follows: stomach (13 of 38, 34%); pancreas (6 of 18, 33%); liver (6 of 18, 33%), and esophagus (4 of 27, 15%; it was less common in tumors of bladder (2 of 19, 10%), kidney (1 of 12, 8%), or breast (1 of 19, 5%), or was not observed at all in brain (0 of 10), lung (0 of 17), head and neck (0 of 10), or ovary (0 of 20) [147]. In endometrial cancer, hypermethylation occurs at an increased frequency, particularly in MSI+ endometrial tumors [148] as well as in cervical cancer, with rates varying from 11 to 94% [133,135,136].

### DNA repair

Alkyating agents induce O6-alkylguanines that can lead to mutations and to cell death unless repaired. The major pathway of repair involves transfer of the alkyl group from DNA to a cysteine acceptor site in the protein O6-alkylguanine-DNA alkyltransferase. Alkyltransferase brings about this transfer without The need for cofactors and DNA is restored completely by the action of a single protein, but the cysteine acceptor site is not regenerated and the number of O6-alkylguanines that can be repaired is equal to the number of active alkyltransferase molecules. A significant fraction of human tumor cell lines do not express the alkyltransferase gene; thus, they are much more sensitive to mutagenesis and killing by alkylating agents [149]. The *MGMT *gene product removes mutagenic and cytotoxic adducts from O(6)-guanine in DNA, the preferred point of attack of many carcinogens (i.e., methylnitrosourea) and alkylating chemotherapeutic agents (i.e., BCNU, temozolamide, etc.). As a consequence, its lack of expression produces opposite effects for cancer development and progression: First, tumors acquire a mutator phenotype characterized by generation of transition point mutations in key genes such as *p53 *and *K-ras*, but at the same time lack of enzymatic activity renders tumors more sensitive to the killing effects of alkylating drugs [150]. While these observations bear clinical and practical implications as predictive or prognostic markers for response in CNS tumors [151], its silencing by hypermethylation can be associated with higher stages, worse survival, or mutations in secondary genes that adversely affect the prognosis of patients with tumors such as gastric, colorectal, head, and neck carcinomas, [152-154] and even in low-grade astrocytomas [155]. There is scarce information concerning the role of *MGMT *gene in cervical cancer; a number of studies have analyzed the frequency of MGMT promoter hypermethylation, which varies from 5–81% [133,134,136,144]. Interestingly, the five cases with *MGMT *or *BRCA1 *methylation did not respond to chemoradiation [133].

### FA-BRCA pathway

Fanconi anemia (FA) is an autosomal recessive chromosomal instability syndrome characterized by hypersensitivity to DNA cross-linking agents and predisposition to cancer, especially leukemia [156]. FA patients are also prone to various solid malignancies, including squamous cell carcinoma. FA is a genetically heterogeneous disease with genes for seven FA complementation (FANC) groups identified [157]. *FANC *genes are essential in DNA repair pathways in normal cellular response to cisplatin and other DNA cross-linking agents. FANC proteins interact with *BRCA *genes in a pathway that involves a number of other genes [158]. Recently, it has been shown that promoter hypermethylation of *FANCF *gene disrupts the FA-BRCA pathway, resulting in cisplatin resistance in ovarian cancer [159]. *FANCF *promoter hypermethylation has also been identified in squamous cell carcinomas of lung and oral cavity [160]. In cervical cancer, a study has shown methylation of BRCA1 in 6.1% [133] of invasive tumors, whereas *FANCF *hypermethylation rate was 30% [161]. Interestingly, hypermetylation of these genes was mutually exlusive in the analyzed cases [161], suggesting the important role of disruption of this pathway for cancer. This abnormality seems to be a late event in cervical carcinogenesis, as no hypermetylation was observed in any case of preinvasive disease [161].

### Mismatch repair

Cells with dysfunction of mismatch repair genes *hMLH1 *and *hMSH2*, as well as *hMSH3*, *hMSH6*, and *hPMS2*, show mutation rates up to 1,000-fold greater than those observed in normal cells [162]. The mutator phenotype, which can be measured by microsatellite instability analysis, has been detected in tumors of patients with hereditary nonpolyposis colorectal sporadic and other types of cancers [163]. Mutations and loss of expression due to gene promoter hypermetylation are the main mechanisms of inactivation of members of this gene family [164]. Hypermethylation and loss of hMLH1 protein expression has been associated with chemotherapy resistance in ovarian and other tumors [165]. The relevancy of this phenomenon has been recently demonstrated by acquisition of hypermethylation of the gene in relapsed ovarian cancer after being treated with chemotherapy, which predicts poor overall survival [166]. Existing data on cervical cancer with regard to hMHL1 expression status and methylation is limited. While some studies have found protein expression loss in invasive lesions [167], others have found the opposite [168], while presence of microsatellite instability appears to correlate with a worse prognosis [169] but not with response to cisplatin in a neoadjuvant setting [170]. Regarding gene promoter methylation, its presence is rare in cervical cancers [134].

## Miscellaneous

### RASSF1A

The Ras Association Domain family 1 (*RASSF1A*) gene consists of two main variants (RASSF1A and RASSF1C), which are transcribed from distinct CpG-island promoters. Aberrant methylation of the RASSF1A promoter region is one of the most frequent epigenetic inactivation events detected in human cancer and leads to silencing of RASSF1A. Hypermethylation of RASSF1A has been observed in a variety of primary tumors including cervix. The product of this gene is involved in several important functions including apoptotic signaling, microtubule stabilization, and mitotic progression and may act as a negative Ras effector inhibiting cell growth and inducing cell death. Its loss of expression in several tumor types is related with worse prognosis [171]. Studies in patients with cervical cancer have demonstrated its silencing by methylation in up to 30% of tumors [172-174,144].

### DAPK

DAP-kinase (*DAPk*) is a Ca(2+)/calmodulin (CaM)-regulated Ser/Thr kinase that functions as a positive mediator of programmed cell death. It associates with actin microfilament and has a unique multidomain structure [175,176]. In cervical cancer, it is methylated in up to 100% of cases [133,135,136,144], which suggests that its loss of expression is needed for cervical cancer progression as seen in an experimental model, in which loss of *DAP-Kinase *expression aids in metastatic potential of lung cancer cells [177].

### TSLC1

The recently identified *IGSF4/TSLC1 *gene codes for an immunoglobulin Ig-like intercellular adhesion molecule that mediates calcium-independent homophilic or heterophilic interactions independently of Ca2. This gene was first identified as a tumor suppressor gene in lung cancer and silencing can derive from loss of heterozygosity or promoter hypermethylation [178]. This gene also has tumor suppressor effects on cervical cancer, as demonstrated by transfection studies in which *IGSF4 *cDNA was introduced into SiHa cells. Transfectants displayed a marked reduction in anchorage-independent growth and when injected into nude mice, these were less able to generate tumors. Progression of the cervical lesion is accompanied by loss of expression of IGSF4. In two studies, it was found that normal epithelium and CIN-1 lesions are free of methylation at *IGSF4*, whereas methylation rate in CIN-III is 35%, which increases to 58 and 65% in invasive tumors [179,180]. These data demonstrate that IGSF4 may have a role in cervical cancer development.

### FHIT

The *FHIT *gene is a tumor suppressor gene located on chromosome 3p14.2 and LOH on the short arm of chromosome 3 and has been detected in up to 75% of cervical carcinomas [181,182]. Many studies have reported altered *FHIT *expression in a variety of carcinomas including head and neck, lung, kidney, gastrointestinal, and breast cancer and in 68% of cervical carcinoma cell lines [183]. Additional studies have confirmed that the *FHIT *gene is abnormally expressed in 30–78% of cervical dysplasia, carcinoma cell lines, and primary tumors [184-188,133,134,136,144]. The antitumorigenic mechanism of *FHIT *is not yet clear; however, for cervical and lung cancer cell lines its reintroduction by use of an adenoviral vector induces strong suppressive effects and reduces tumorigenicity due to an apoptotic effect associated with caspase-8 cleavage and activation of the effector caspase-3 [189]. Hypermethylation of this gene has been associated with loss of expression and advanced stages of cervical carcinoma, suggesting its participation in disease progression [190].

### HIC1

*HIC1 *(hypermethylated in cancer) is a tumor suppressor gene unique in the sense that it has never been found mutated but is found silenced by hypermethylation. This gene encodes a zinc-finger transcription factor that belongs to a group of proteins known as the POZ family. HIC1 is hypermethylated and transcriptionally silent in several types of human cancer. Homozygous disruption of Hic1 impairs development and results in embryonic and perinatal lethality in mice, while heterozygous mice develop many different spontaneous malignant tumors including a predominance of epithelial cancers in males and lymphomas and sarcomas in females. Complete loss of Hic1 function in heterozygous mice appears to involve dense methylation of the remaining wild-type allele promoter [191]. It has recently been shown that its loss of function accentuates the tumorigenic effect of loss of p53 [192]. It has been found that the *HIC1 *gene is down regulated in many cervical cancer cell lines and re-expressed upon treatment with a demethylating drug [133]. In primary cervical tumors, its methylation rate varies between 18 and 45% [133,135]. These results support the tumor suppressor role of *HIC1 *and its inactivation by promoter methylation in cervical cancer.

### RARβ2

Retinoic acid is essential for regulation of epithelial cell differentiation. The intracellular effects of RA are mediated by RA-binding nuclear receptors, including RA receptors alpha, beta, and gamma. Ligand-activated receptors induce transcription of target genes by binding to RA-responsive elements in promoter regions. One target gene is the RAR beta gene, which encodes a potential tumor suppressor. Complete or partial inhibition of gene expression has been observed in many tumor cell lines and in primary human tumors [193]. A well-known mechanism of RAR-β2 inhibition demonstrated in breast and colon carcinomas is hypermethylation of its promoter [194]. In cervical cancer, the RARβ2 gene is of particular interest because retinoic acid inhibits transformation of human keratinocytes by HPV-16 [196] and leads to regression of moderate cervical dysplasia [197]. In addition, RA in combination with interferon alfa is a highly effective antitumor therapy for patients with cervical cancer [198]. However, the existence of intrinsic or acquired resistance to retinoic acid is well-established [199]. Rate of RAR-β2 methylation progressively increases from 11% in low-grade to 29% in high-grade lesions and from 33–63% in invasive cancers [133,136,200,201], suggesting that this abnormality is an early event in multistage cervical carcinogenesis.

### Tissue inhibitors of metalloproteinases

*TIMP-2 *and *TIMP-3 *are endogenous inhibitors of matrix metalloproteinases that possess growth promoting effect in cell culture, and anti-tumoral, anti-apoptotic, and anti-angiogenic effects in animal model systems *in vivo*. It has been shown that these endogenous inhibitors are downregulated by methylation in cervical carcinomas in a variable proportion of patients [144,202,203] and may contribute to progression of cervical cancer.

### Caveloin

*Caveolin-1 *is commonly downreglulated in cervical cancer cells and its introduction via gene transfer restores caveolin-1 protein expression and impairs growth in SiHa cells, which supports its role as a tumor suppressor gene [204]. Although it is known that HPV-E6 oncoprotein reduces caveolin expression, in a small percentage of cervical cancer tumors silencing occurs via promoter methylation [205].

### Global hypomethylation

As stated previously, tumor cells may have up to 60% less global DNA hypomethylation than their normal counterparts [77,78] and interestingly, DNA hypomethylation is progressive from premalignant conditions to fully developed malignancies [80]. The main mechanisms set forward in attempting to explain cancer causation by hypomethylation are chromosome instability and reactivation of transposable elements and/or inappropriate gene activation [81]. Analyses of global DNA methylation have been performed in samples covering the full spectrum of cervical lesions. Kim et al., analyzed 41 samples from colposcopically identifiable lesions for methylation by incorporation of [3H]-S-adenosylmethionine. As expected, the extent of 3H-methyl group incorporation was increased three- and seven-fold in DNA from cervical dysplasia and cancer as compared with DNA of normal cervices and within dysplasias as long as they progress from normal to low- and high-grade [206]. Subsequently, this group performed a similar analysis in 83 cases and obtained essentially the same results with regard to DNA hypomethylation, highlighting the fact that lower methylated DNA correlated wtih serum folate levels [207]. Using a computer-assisted assay based on quantitative analysis of DNA methylation in individual interphase nuclei by immunolabelling with anti-5-methylcytosine antibodies, progressive hypomethylation was observed in dysplastic and cancer cells as compared to normal controls [208]. These data, along with observations of gene promoter hypermethylation of a number of genes during pre-invasive to invasive stages of cervical cancer, demonstrate that both phenomena are coincident during carcinogenesis of the cervix uteri.

## Histone alterations in cervical cancer

At the chromatin level, there are some indications that the pattern of chromatin distribution in smears may aid in diagnosis of cervical neoplasia, particularly for glandular lesions [209], but the molecular bases for these chromatin alterations have yet to be determined. Contrariwise, histone changes at a global level in cancer and normal cells have only recently been studied [210]. A recent report showed that during the tumorigenic process, cancer cells had a loss of monoacetylated and trimethylated forms of histone H4, predominantly at acetylated Lys16 and trimethylated Lys20 residues of histone H4, which were associated with hypomethylation of DNA repetitive sequences, a hallmark of cancer cells [211]. In this line, tumor cell acetylation level and methylation of histones in prostate carcinoma cells identified two disease subtypes with distinct biological behaviors in patients with prostatic carcinoma [212]. In cervical cancer, it has been reported that phosphorylated and acetylated forms of histone H3 in cytologic smears shows a marked association of histone H3 modifications with progression of the disease from CIN I to CIN II and CIN III [213].

The balance between histone deacetylases and histone acetyl transferase activities is a major player in regulation of gene transcription [214]; hence, this balance must be mantained in normal cells, or otherwise unchecked cell proliferation and cell death would occur. E6 and E7 oncoproteins of HPV target numerous cellular proteins to disturb cell growth and proliferation [30] including HDACs and HATs. The HPV-E7 protein from high-risk types [215] binds to HDACs, and this interaction occurs through an intermediary protein called Mi2β, a member of the nucleosome remodeling and histone deacetylation (NURD) complex that possesses the ability to modify chromatin structure through both deacetylation of histones and ATP-dependent nucleosome repositioning. Binding of HDACs to E7 is independent of binding to Rb, and mutations on E7 abolishes its binding to HDAC1 and results in a loss of the ability of E7 to efficiently transform rodent fibroblasts [216]. E6 protein of HPV high-risk types also shares, with other small DNA tumor viruses, the capacity for targeting CBP/p300 in an interaction involving the C-terminal zinc finger of E6 and CBP residues 1808–1826 to downregulate p53 transcriptional activity independently of removal of cellular p53 protein through the proteosome degradation pathway [217]. As for E7, binding of E6 to the transcriptional co-activator p300/CBP is essential for cell transformation [218].

## Loss of imprinting

Imprinting refers to the condition established during gametogenesis, dictating that a specific parental allele is or is not expressed in the offspring. Loss of imprinting is implicated not only in developmental disorders such as Prader-Willi syndrome (PWS) and Angelman syndrome (AS), but also in cancer [219]. In Wilms tumors, loss of imprinting leads to biallelic expression of *IGF2 *and reciprocal loss of expression of *H19*, a non-transcribed RNA with tumor suppressor activities, while IGF2 promotes tumor formation by inhibiting apoptosis [220,221]. In cervical cancer, a sole study has found a high frequency of both loss of heterozygosity and loss of imprinting for H19 and IGF2 genes, suggesting that they participate in the molecular pathogenesis of cervical cancer [222].

## Translational opportunities of epigentic alterations in cervical cancer

Identification of numerous epigenetic alterations of cervical cancer in all stages of the disease process offers new possibilities of diagnosis and treatment of cervical cancer (Table 3).

**Table 3:** Translational opportunities from epigenetic alterations in cervical cancer

Early detection	Identification of a set of hypermethylated genes from cytological smears
Prognostic/predictive	Determination of global methylation or histone modifications in tumor or peripheral blood cells Determination of hypermethylated gene promoters from serum/plasma DNA
Therapy	DNA methylation inhibitors and/or HDACs inhibitors alone or as chemo- or radio-sensitizers

## Early detection

Cervical cancer remains a model disease for screening due to its long natural history and ease of sampling and reading cytologic abnormalities; dramatic reductions in mortality from this neoplasia achieved in countries with well-organized detection programs validate this fact [223]. Nevertheless, the test as such has low sensitivity, though high specificity for detecting CIN-3 [224]. More recently, testing for high-risk types of HPV infection are in use to aid in the triage of women with atypical squamous cells of undertermined significance; nonetheless, the large number of women requiring additional testing and the inability of cytology or HPV testing to identify women at higher risk for disease progression impose greater efforts on health systems. Thus, novel screening alternatives are needed. The realization that genetic and epigenetic alterations are present at the earliest steps of the malignant progression of cervix uteri has led to testing the presence of these abnormalities, such as p16 expression [225,226]. A large number of studies looking at the methylation status of tumor suppressor genes have uncovered that some genes are found hypermethylated in preinvasive lesions, raising the possibility that testing for methylation of either of these or a set of these may prove to be a useful screening tool [133-136]. However, there is limited information with respect to the sensitivity and specificity of methylated genes for identification of women with cervical dysplasia and cancer as well as comparisons of results using different sources of samples, either exfoliated cells or paraffin-embedded fresh biopsy samples. In this regard, a very comprehensive study investigated the methylation profile of 20 genes (*p16, p15, CCND2, RASSF1, RARb, TWIST1, SYK, HIC1, VHL, PRDM2, SFN, MLH1, MGMT, APC, CDH1, and CDH13*) in exfoliates and biopsies of 319 women that participated in a cytology screening study. By logistic regression, the authors determined the best set of candidate genes for employment as a disease markers. First, they found similar detection rate of methylation regardless of sample source, and second, they found that *CDH13, DAPK1, RARb*, and *TWIST1 *were the genes showing statistically signficant increase with lesion severity, and *DAPK1, RARb*, *and TWIST1*, the best panel of hypermethylated genes. At least one of the three genes was hypermethylated in 57% of samples with CIN-3/CIS and in 74% with invasive cancer, but in only 5% of samples with CIN-1. Estimated specificity of the panel was 95% with sensitivity of 74% (95% confidence interval [CI 95% ], 73–75%) for invasive carcinoma and 52% (95%,49–55%) for CIN-3/CIS. These findings provide preliminary evidence on the potential usefulness of a panel of genes to be tested for hypermethylation in cytology samples; however, additional studies are needed before this epigenetic-based screening test could be adopted [227].

## Methylated genes in serum of patients with cervical cancer as biomarkers

Circulating nucleic acids represent a biomarker that might be used in early detection of cancer, in the follow-up of patients with cancer or as a prognostic factor. Presence of nucleic acids in plasma or serum of patients with cancer has been recognized since the 1970s, but it was not until 1989 that the neoplastic characteristics of plasma DNA in patients with cancer were recognized [228], and this was followed by detection by PCR-based techniques of specific gene mutations present in the primary tumor, such as *Ras *family members in tumors such as pancreas and myelodysplastic syndromes [229,230]. Within this field of study, cell-free circulating RNA and nucleosomal DNA have also been studied in patients with cancer [231,232]. The feasibility of applying a PCR-based technique such as the Methylaton-Specific PCR (MSP) [233] for amplification of gene promoters from DNA circulating in serum or plasma paved the way for the study of epigenetic alterations not only in serum plasma of patients with cancer, but also in other biologic fluids, such as urine and sputum [234,235].

The methylation status of several genes present in the serum or plasma of patients with cervical cancer has been studied with regard to their prognostic signficance. In a study analyzing serum samples of 93 patients with the methyLight technique for gene promoter methylation of *CALCA, hTERT, MYOD1, PR*, and *TIMP3 *genes, methylation rates were 62, 0, 25, 79, and 4% respectively, and in all but one case corresponding primary tumors also had these genes methylated. When authors looked at survival, methylation of *MYOD1 *was strongly associated with shorter, disease-free and overall survival with median survival of 1.9 and 6.1 years for methylated and unmethylated cases, respectively [203]. The same group of researchers reported on the prognostic significance of *CDH1 *(E-cadherin) and CDH13 (H-cadherin) using the same technique and the same number of patients. The main finding was that aberrant methylation of either *CDH1 *or *CDH13 *was found in 40 (43%) patients, and median survival for these patients with methylated genes was 1.2 vs. 4.3 years in *CDH1 *or *CDH13 *methylation-free patients [143]. Data suggesting that methylation of gene promoters in patients with cervical cancer is a common phenomenon were reported by another group that analyzed DAPK, p16, and MGMT genes; these authors also found a strong correspondence between methylation in serum and in primary tumors with methylation frequencies in serum of 40, 10, and 7.5% of these genes, respectively [236]. Together, these data encourage further studies to find a set of methylated genes that would have prognostic significance but that would also serve as surrogate markers of efficacy of epigenetic therapies.

## Treatment

### Demethylating agents

Reactivation of tumor suppressor genes that have been silenced by a epigenetic mechanism such as gene promoter methylation is a very attractive molecular target for cance therapy. Inhibitors of DNA methylation have demonstrated the ability to inhibit hypermethylation, restore suppressor gene expression, and exert antitumor effects in *in vitro *and *in vivo *laboratory models. There are several demethylating agents currently being evaluated in preclinical and clinical studies (Table 4). The classical demethylating agents comprise the analogs of deoxycytidine:5-azacytidine, 5-aza-2-deoxycytidine, 1-β-D-arabinofuranosil-5-azacytosine, and dihydro-5-azacytidine. 5-azacytidine and its analog are the most studied and were developed over 30 years ago as classical cytotoxic agents, but were subsequently discovered to be effective DNA methylation inhibitors [237]; these were tested as such in several phase II studies against solid tumors demonstrating very modest activity [238]. To the contrary, their antileukemic activity was very promising and both are being revived as a consequence of their demonstrated inhibitory activity upon DNA methylation and gene-reactivating function. Currently, 5-azacytidine is Federal Drug Administration (FDA)-approved for use against myelodysplastic syndrome, and the hydrosoluble analog 5-aza-2-deoxycytidine is being tested in a variety of solid tumors as DNA demethylating agent [239]. Despite the poor activity against solid tumors of these nucleoside analogs, it is remarkable that the proof of the concept iwith regard to the ability of demethylating compounds to demethylate and reactivate tumor suppresor gene expression has been demonstrated in solid tumors [136,240]. Whether or not the reactivating effect can translate into a clinical response on its own or in combination with classical cytotoxic therapies remains to be demonstrated.

**Table 4:** Epigenetic therapy agents

**DNA methylation inhibitors**
*deoxycytidine analogs:* 5-azacytidine, 5-aza-2-deoxycytidine, 1-β-D-arabinofuranosil 5-azacytosine, dihydro- 5-azacytidine
*nucleic acid-based:* MG98 antisense oligonucleotide
*cytidine deaminase analogs:* zebularine
*non-nucleoside analogs:* (-)-epigallocatechin-3-gallate, procaine, procainamide, hydralazine

**Histone deacetylase inhibitors**
*Small molecular weight carboxilates:* sodium butyrate, valproic acid, sodium phenylbutyrate and pivaloyloxymethyl butyrate
*Hydroxamic acids:* SAHA, trichostatin A, SBHA
*Benzamides:* CI-994, MS-275
*Epoxyketones:* trapoxin B, 2-amino-8-oxo-9,10-epoxydecanoic acid
*Cyclic peptides:* apicidin, depsipeptide
*Hybrid molecules:* CHAP 31, CHAP50

As a second category of demethylating agents, we note the antisense oligonucleotide MG98 against the 3' untranslated region of *DNMT1 *mRNA, which codes for the enzyme DNA methyltransferase 1 that is responsible for maintenance of DNA methylation [241]. This agent has shown an ability to inhibit DNMT1 expression without affecting other *DNMTs*, and to cause demethylation with re-expression of p16 in bladder and colon cancer cell lines as well as to produce tumor growth inhibition in nude mice bearing human lung and colon xenografts [242]. MG98 has been evaluated in a phase I trial in patients with advanced or refractory solid tumors and has demostrated its tolerability despite the fact that dose-limiting toxicities of transaminitis, thrombocytopenia, and fatigue prohibited higher doses. Its molecular efficacy was demonstrated by producing a decrease in *DNMT *mRNA levels in six of 10 patients [243].

The fact that deoxycytidine analogs such as current cytotoxic agents are not only carcinogenic but also exhibit neutropenia as their dose-limiting toxicity even when used at doses required for demethylation [244] has renewed interest in finding effective and less toxic demethylating agents. Zebularine is a new oral cytidine analog originally synthesized as a cytidine deaminase inhibitor that has been shown to cause demethylation and reactivation of a silenced and hypermethylated *p16 *gene in human bladder tumor cells grown in nude mice. Zebularine was also demonstrated as minimally cytotoxic *in vitro *and *in vivo *and can be given continuously at a lower dose to maintain demethylation for a prolonged period, only possible because of its low-toxicity profile; to date, there are no results of clinical trials with this agent [245]. Within this class of so-called "non-toxic and orally administered agents" there is the green tea major polyphenol (-)-epigallocatechin-3-gallate (EGCG), which resulted as an effective inhibitor of DNMT activity at micromolar concentrations and that was able to demethylate and reactivate expression of several tumor suppressor genes such as p16, RAR-β2, and MGMT in cancer cell lines [246]. There is another class of so-called "old drugs" whose demethylating activity upon gene promoters of tumor suppressor genes was recently highlighted. Procainamide, a nonnucleoside inhibitor of DNA methyltransferases approved for treatment of cardiac arrhythmias, can demethylate the *GSTP1 *promoter, a common somatic genome change in human prostate cancer and reactivates *in vitro *and in nude mice the expression of the gene [247]. A related drug, procaine, has also the ability of demethylating and reactivating tumor suppressor gene expression, such as the *RAR*β*2 *gene in a breast cancer cell line effect that is accompanied by growth-inhibitory actions [248]. Our group has recently shown *in vitro *and *in vivo *promoter demethylation and tumor suppressor gene transcriptional reactivation mediated by the antihypertensive compound hydralazine, a well-tolerated drug devoid of the common side effects of cytotoxic chemotherapy agents [249]. Its DNA demethylating activity can be explained by the interaction between its nitrogen atoms with residues Lys162 and Arg240 of the DNA methyltransferase active site, as shown in a silico modeling [250].

### Histone deacetylase inhibitors

HDACs are seen as a potential target for cancer treatment. HDAC inhibition has been reported to induce tumor cell differentiation, apoptosis, or growth arrest, depending on the experimental system [251,252], and to sensitizer cells to chemotherapy [253] or radiation therapy [254] However, the HDAC-dependent mechanisms accounting for the observed and rather selective modulation of gene expression, as well as specific patterns of antitumor activity, remain poorly understood.

Currently, there are six structurally distinct classes of HDAC inhibitors at diverse stages of preclinical and clinical development (Table 4); these act by binding to various portions of catalytic domains within class I and II HDACs: 1) *Small molecular weight carboxilates *(sodium butyrate, valproic acid, sodium phenylbutyrate and pivaloyloxymethyl butyrate); 2) *hydroxamic acids *(suberoylanilide hydroxamic acid -SAHA-, trichostatin A, suberic bishydroxamic acid -SBHA-, and others); 3) *benzamides *(CI-994 and MS-275); 4) *epoxyketones *(trapoxin B, HC-toxin, or 2-amino-8-oxo-9,10-epoxydecanoic acid); 5) *cyclic peptides *(apicidin, depsipeptide), and 6) *hybrid molecules *CHAP 31 and CHAP50) [255].

In general, HDAC inhibitors are at most at the early stages of clinical development. Among them, SAHA has shown in a recently reported phase I study of 73 patients with advanced cancer a complete response in a patient with transformed diffuse large B-cell lymphoma for 17 months, three partial responses in B-cell lymphoma, laryngeal cancer, and papillary thyroid cancer, and prolonged stabilization in patients with renal carcinoma [256]. In another study of MS-275 in 31 heavily pre-treated patients despite the fact that no objective responses were observed, 15 cases achieved disease stabilization for 62–309 days [257]. In another phase I study with CI-994, one of 42 patients with a pre-treated lung adenocarcinoma showed a partial response over 2 years, whereas three additional patients with small-cell lung cancer, colorectal, and renal carcinoma had stable disease [258]. In a phase II study of pivaloyloxymethyl butyrate in pre-treated non small-cell lung cancer, three (6.4%) of 47 patients achieved partial responses and 14 (30%) patients achieved stable disease [259].

The strong interplay between DNA hypermethylation and histone deacetylation for silencing and modulating the expression of a number of cancer-related genes predicts not only a synergy in gene expression at global [260] and individual gene levels [261] but also in antitumor activity. For instance, combinations of decitabine with trichostatin A or depsipeptide synergistically reactivate silenced tumor suppressor genes including *MLH1*, *TIMP3*, *CDKN2B*, *CDKN2A*, *ARHI*, *gelsolin*, and *maspin *[262-264] and increase the level of tumor cell apoptosis [265]. Thus, a logical step forward is to combine a demethylating with a histone deacetylase inhibitor for cancer treatment.

## Epigenetic therapy in cervical cancer

Despite ample experimental evidence supporting the development of drugs that target the epigenoma via inhibition of DNA methylation or histone modification as cancer therapy, clinical results are pending to date. At present, the majority of the information has been generated in hematologic neoplasms and advanced or refractory solid tumors. The main findings of this form of therapy in cervical cancer are depicted in Table 5.

**Table 5:** Main findings of epigenetic therapy in cervical cancer

Lack of response to DNA methylation inhibitor 5-aza-2-deoxycytidine as a single agent in advanced or recurrent cervical cancer*

Response rate of 38.1% with the DNA methylation inhibitor 5-aza-2-deoxycytidine plus cisplatin in advanced or recurrent cervical cancer*

Demethylation and reactivation of the expression of several tumor suppressor genes in the tumors of cervical cancer patients in a phase I trial of the DNA methylation inhibitor hydralazine

Sustained stabilization of disease in a patient with cervical cancer treated within a phase I trial of the HDAC inhibitor MS-275

Major response in a patient with cervical cancer being treated with the HDAC inhibitor valproic acid followed by a single dose of epirubicin within a phase I trial

H3 and H4 hyperacetylation as well as inhibition of deacetylase activity in the tumors of cervical cancer patients with cervical cancer in a phase I trial of the HDAC inhibitor magnesium valproate

Ongoing phase II trial of the combination of hydralazine and magnesium valproate added to cisplatin chemoradiation in FIGO stage IIIB patients

*DNA methylation was not analyzed in these trials.

Decitabine was used against advanced or recurrent cervical carcinoma in a small phase II study at a time when it was already known that this drug was a demethylating agent. The schedule used was 75 mg/m^2 ^per hour 3 times on day 1 at 7-hours intervals and repeated every 5 weeks. None of the 14 patients evaluated for response responded or even had disease stabilization, and one patient died from neutropenic sepsis [266]. Aparicio et al. reported a phase I study using 5-aza-2'-deoxycytidine in patients with advanced solid tumors using escalating doses of 20, 30, and 40 mg/m^2 ^employing a 72-hour continuous infusion every 28 days. Quantitative Methyl-Light reaction was used to evaluate changes in promoter methylation in 19 genes, but no consistent evidence of gene demethylation was documented despite the fact that grade 4 neutropenia was found in nearly one third of the patients. This latter finding argues against its use as a DNA demethylating agent in solid tumors because despite such toxicity, steady-state levels reached during the study (0.1–0.2 μM) are below levels needed *in vitro *to demethylate gene promoters [244]. On the other hand, a number of genes showed increased methylation, which could be derived from the cytotoxicity of this nucleoside analog. It is well-known that the majority of cytotoxic agents lead to increase in DNA methylation *in vitro *and in cancer patients [267]. In an attempt to exploit the synergism observed between decitabine and cisplatin, a phase II study with this combination was performed in advanced or recurrent cervical cancer. Initial dose of cisplatin was 40 mg/m^2^, whereas decitabine dosage was 50 mg/m^2 ^for 3 consecutive days every 21 days. Due to toxicity, the cisplatin dose was reduced to 30 mg/m^2^. Twenty five patients were included in the study; 24 were eligible for evaluation of toxicity, whereas 21 were eligible for evaluation of tumor responses. A total of 75 cycles of chemotherapy were administered to patients, with a median of three cycles (range: 1–8 cycles per patient). The most frequently observed side effect was grade III and IV neutropenia in 68.0% of cases; one patient died of complications caused by drug-related neutropenic sepsis. Of a total of 21 patients evaluable for tumor response, eight (38.1%) achieved a partial response, whereas stable disease was documented in five cases (23.8%); median survival was 5 months [268]. DNA methylation at global or individual gene was not evaluated; thus, the merit of this combination as DNA methylation-targeted therapy remains to be established. Among other DNA demethylating agents, in a phase I study hydralazine was administered to cohorts of four patients at the following dose levels: 1) 50 mg/day; 11) 75 mg/day; III) 100 mg/day, and IV), 150 mg/day. Tumor biopsies and peripheral blood samples were taken the day before and after treatment to analyze by MSP and RT-PCR the genes *APC, MGMT; ER, GSTP1, DAPK, RARβ, FHIT*, and *p16 *pre- and post-treatment for DNA promoter methylation and gene expression. The drug was well-tolerated and rates of demethylation at the different dose levels were as follows: 50 mg/day, 40%; 75 mg/day, 52%; 100 mg/day, 43%, and 150 mg/day, 32%; nine of 12 informative cases (75%) re-expressed the gene. Interestingly, there was neither change in methylation status of H19 and clone 1.2 nor changes in global DNA methylation in peripheral blood mononuclear cells [136].

With regard to histone deacetylase inhibitors for cervical cancer, limited clinical information obtains from a phase I study of MS-275; a patient with cervical cancer had a sustained period of 10 months of stable disease [257], supporting the potential activity of this class of drugs for this tumor type. Also encouraging are recent data from a phase I study for metastatic solid tumors in which valproic acid was administered as an IV loading dose followed by five oral doses were administered every 12 hours followed by a dose of epirubicin at day 3. At the time of reporting, 16 patients have been treated at four dose levels: VPA 15, 30, 45, and 60 mg/kg, with epirubicin at 75 mg/m^2^. The maximum tolerated dose has not been reached and dose escalation is continuing. Major responses were observed in all tumor types including in anthracycline failures and in anthracycline-resistant cancers such as melanoma and cervical carcinoma, suggesting that inhibition of HDAC activity may chemosensitize tumor cells [269]. Use of valproic acid in the form of magnesium valproate was recently reported in a phase I study where 12 newly diagnosed patients with cervical cancer were treated after a baseline tumor biopsy and blood sampling at the following dose levels (four patients each): 20 mg/kg; 30 mg/kg, or 40 mg/kg for 5 days via oral route; at day 6, when steady state of valproic acid was achieved, tumor and blood sampling were repeated. All patients completed the study medication and mean daily dose for all patients was 1,890 mg with depressed level of consciousness grade 2 as most frequent toxicity. After treatment, there was hyperacetylation of H3 and H4 in tumors of nine and seven patients, respectively, whereas six patients demonstrated hyperacetylation of both histones, and tumor deacetylase activity decreased in eight patients (80%). These data demonstrate for the first time that magnesium valproate at a dose between 20 and 40 mg/kg inhibits deacetylase activity and hyperacetylates histones in malignant solid tumors [270]

It is remarkable that DNA methylation inhibitors such as decitabine [271] and zebularine [272] as well as HDAC inhibitors including SAHA, M344, depsipeptide [273], and valproic acid [274] are radiosensitizers. In addition, it is very noteworthy that HDAC inhibitors such as phenylbutyrate, trichostatin A, and valproic acid are able to reduce cutaneous radiation toxicity following radiotherapy [275]. Therefore, radiation or chemoradiation in combination with DNA demethylating agents and/or HDAC inhibitors is a research avenue to be explored. Based on these data, a phase II study of transcriptional therapy with the demethylating hydralazine plus the histone deacetylase inhibitor magnesium valproate added to chemoradiation with cisplatin is ongoing in FIGO stage IIIB cervical carcinoma.

## Conclusion

Epigenetic alterations are at least if not more important than genetic defects for the development and progression of malignant diseases. In cervical cancer, a number of epigenetic alterations occurring during all stages of cervical carcinogenesis in both human papillomavirus and host cellular genomes have been identified. These include global DNA hypomethylation, hypermetylation of key tumor suppressor genes, and histone modifications. From the diagnostic point of view, because epigenetic abnormalities occur very early in the carcinogenic process they can potentially be exploited as molecular markers for early detection. In this sense, identification of a set of genes hypermetylated in cytologic smears could offer novel means for screenning. Assessment of hypermethylated genes in primary tumor or in serum DNA may serve as a prognostic factor or as a means of predicting response to radiation, chemotherapy, and transcriptional agents.

In the therapeutic field, transcriptional therapy is a very promising form of cancer treatment that is being extensively evaluated. It is too early to evaluate usefulness. However, it has now been demonstrated that inhibitors of DNA methylation and histone deacetylases can reactivate expression of tumor suppressor genes and induce hystone hyperacetylation in the tumors of patients with cervical cancer after treatment with these agents.

## Authors' contributions

AD-G conceived and wrote the manuscript; ML contributed with the HPV section; MC, LC, CA and EC participated in the discussion, analysis of the content.
